# Genetic Heterogeneity and Clonal Evolution of Tumor Cells and their Impact on Precision Cancer Medicine

**DOI:** 10.4172/2329-6917.1000124

**Published:** 2013-11-18

**Authors:** Hatem E Sabaawy

**Affiliations:** 1 Department of Medicine, Rutgers-Robert Wood Johnson Medical School, USA; 2 Department of Cellular and Molecular Pharmacology, Rutgers-Robert Wood Johnson Medical School, USA; 3 Rutgers Cancer Institute of New Jersey, New Brunswick, NJ 08903-2681, USA

**Keywords:** Genetic heterogeneity, Clonal evolution, Precision medicine

## Abstract

The efficacy of targeted therapies in leukemias and solid tumors depends upon the accurate detection and sustained targeting of initial and evolving driver mutations and/or aberrations in cancer cells. Tumor clonal evolution of the diverse populations of cancer cells during cancer progression contributes to the longitudinal variations of clonal, morphological, anatomical, and molecular heterogeneity of tumors. Moreover, drug-resistant subclones present at initiation of therapy or emerging as a result of targeted therapies represent major challenges for achieving success of personalized therapies in providing meaningful improvement in cancer survival rates. Here, I briefly portray tumor cell clonal evolution at the cellular and molecular levels, and present the multiple types of genetic heterogeneity in tumors, with a focus on their impact on the implementation of personalized or precision cancer medicine.

## Introduction

The lifetime risk of a clinical cancer diagnosis in humans is around one in three, with more than 10 million cases diagnosed each year [1]. The oldest description of cancer dates back to Ancient Egypt around 1600 BC when a number of breast cancer patients were described to be surgically treated by cauterization. Since then, surgical resection and adjuvant therapy can cure well-confined primary tumors, however, metastatic disease is largely incurable because of its systemic nature and resistance to existing therapies. Currently, cancer is a leading cause of death globally, and more than 90% of mortality from cancer is attributable to metastasis, not the primary tumors from which these lesions arise. In the case of leukemias, once leukemic cells are less confined to the bone marrow or the thymus, and are found in the peripheral blood, the disease is already a systemic disease. Despite the significant investment in cancer research and clinical trials over several decades around the world and in the US, especially after enabling the National Cancer Act in 1971, only few targeted therapies in leukemias and some solid tumors deemed therapeutically effective in phase III trials, and most current advanced cancer therapies have marginal improvement in survival. A better understanding of tumor development and better classification of tumor types at the cellular and genetic levels might provide improved strategies to suppress progression of prenoplastic lesion towards the malignant and the metastatic state(s) and offer more specific targets for drug development that would lead to more effective and personalized cancer therapy.

It has been known that a large number of patients treated for cancer don’t respond to therapy given to them. This indicates that every drug does not work similarly in every patient, given that every patient has a unique biology and unique tumor architectures. These variations should be reflected in their choice of therapy to improve efficacy and minimize side effects. Several molecular mechanisms have been implicated in the development of neoplastic lesions and therapy resistance, and novel targeted agents to treat these neoplasms after diagnosis and/or relapse have been developed. However, variable efficacy has been observed in late-stage clinical trials, most likely because of the lack of complete understanding of the tumor development process and the biological heterogeneity of these tumors. The key response to the long-term disappointments in the fight against cancer must be revolutionary and lies in implementation of personalized or precision medicine where cancer therapy is tailored to each patient’s biology and tumor signatures to achieve the best medicinal outcome for that individual.

Precision cancer medicine traditionally involves determining the biological status of an individual tumor before therapy by assessing genetic signatures, hormone metabolism, and signaling activity, and then directing tailored treatment accordingly. The recent surge in Next Generation Sequencing (NGS) of cancer genomes has supported the expansion of molecular cancer profiling to support precision cancer medicine. However, translation of these genetic and metabolic findings into clinically valuable genetic, epigenetic, proteomic, biochemical, metabolic and imaging biomarkers for diagnosis, prognosis, and response to therapy is an extended intricate process that remains critical for the wide implementation of precision medicine in cancer therapy. The remaining central challenges for this approach include selection of optimal drug targets, evaluation of genetic profiles and genetic interactions, determining proper combinations of therapies, implementing clinical platforms in phase I studies, and resolving the organizational, commercial, regulatory, and societal challenges facing these precision cancer medicine approaches. To name a few, organizational challenges include structure and administration of personalized clinical trials, commercial concepts of personalized therapy such as pay for performance of multiple tailored treatments replacing blockbuster drugs, regulatory evaluation of outcome of personalized medicine, ethical considerations of genetic testing, and level of acceptance of cancer patients to the new paradigm of personalized medicine studies. Ultimately, a better understanding of the tumor and metastatic developmental process, and an optimum design of targeted tailored therapies are instrumental in the success of precision cancer medicine. Two concepts have recently gained a great deal of attention, and remain at the center stage of our understanding of tumor development for designing better-tailored therapies; these are tumor cell clonal evolution and tumor heterogeneity.

## Tumor Cell Clonal Evolution

**Figure 1:**
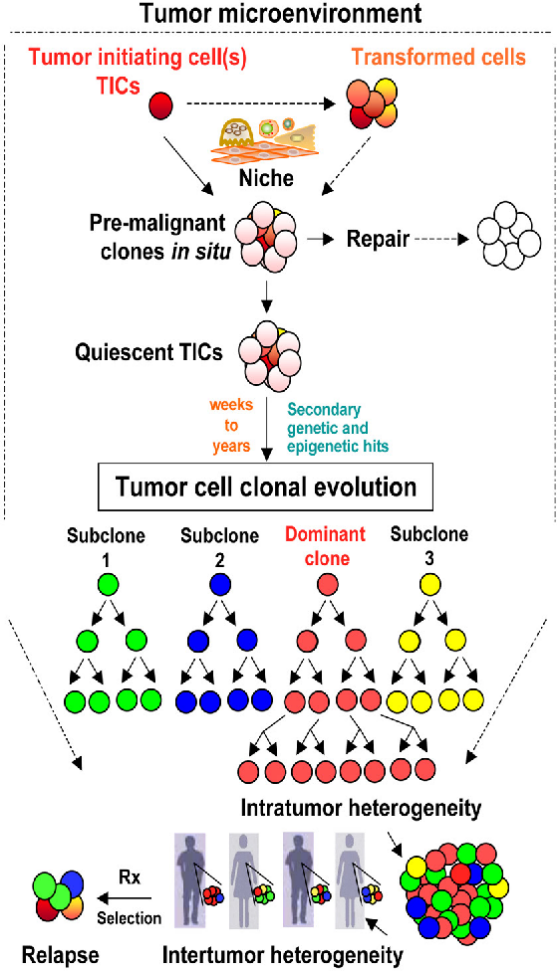
Tumor intiating cells, tumor cell clonal evolution, and tumor heterogeneity A simplified diagram displaying the different tumor evolution processes under review. Within the tumor microenvironment, Tumor intiating cells (TICs) initiate premalignant clones through the interactions with the niche cells. The premalignant clones may generate frequent sublinical lesions such as carcinoma in situ (or preleukemic conditions) that are either in most cases repaired at the cellular levels through cell death mechanisms, and at the genetic levels through DNA repair of driver genetic mutations. The premalignant clones may alternatively remain harboring quiescent TICs that can then undergo tumor cell differentiation and/or plasticity within the competitive clonal evolution process to compete for space, nutrients, and proximity to vascular supply. Acquisition of secondary genetic and epigenetic changes in TICs or supportive tumor cells that favor enhanced self-renewal and clonal growth allows premalignant lesions to become a clinically diagnosed malignancy. This tumor evolution process can take from weeks to several years or even decades depending on the tumor type and the the host genetic and enviromental exposure factors. TICs are the units of clonal evolution and their diversity seed the recently identified tumor heterogeneity within each tumor (intratumor heterogeneity) (represented by a dominant clone in red, and three additional subclones in green, blue and yellow in the model). I proposed one dominat clone and three subclones for simplicity. Indeed, the frequency of subclones can be unlimited and dependes on the sensetivity of the detection assays. Neverthless, only detectable clones are thought to have clinical implications. Genetic and phenotypic variations are also detected between individuals with the same tumor type (Intertumor heterogeneity), and occur due to the diversity of clones generated during tumor cell clonal evolution. Upon treatment (Rx), relapse might occur from the diagnostic dominant clone that acquired more selective and drug resistance features, or from sublones that have acquired or inherited resistance to therapy. Therefore, detecting clonal heterogeneity and mechanisms of development of therapy resistant clones is critical for tailoring combination therapies for personalized cancer medicine. Solid arrows indicate defined pathways while dashed arrows indicate suggested mechanisms.

NGS for cancer samples is now widely accessible, increasingly affordable, and provides a transformative influence on cancer care with a particular insight into the complexity of the cancer genome [2]. With it, we came to realize the true meaning of the statement that cancer is a disease of the genome. Neoplasms in general represent abnormal outgrowth of tumor cells that gain selective advantages in cell growth, survival, and metabolism. Their sustained growth kinetics lead to the formation of dominant neoplastic clones that compete with, and override normal and preneoplastic cells for space, energy, and nutrient requirements utilizing their genetic and non-genetic drivers for selective advantage. Sequencing of genomes from tumor cells within these clones revealed that tumors have partially or fully transformed cells that harbor hundreds to thousands of genetic mutations, chromosomal alterations, and epigenetic aberrations. The majority of these mutations represents neutral (passenger) mutations, while the selection and propagation of dominant clones of tumor cells that ultimately lead to malignant transformation are both successively and may be independently sustained by multiple different combinations of driver mutations. The orchestrated and sustained signaling actions of these driver mutations during the process of clonal evolution provide, at each stage, a selection advantage, and allow dominant tumor cell clones to finally control various interactions with microenvirmental clues at the eminent stages of tumor development (Figure 1) [3,4]. Intrinsic changes in Tumor Initiating Cells (TICs) result partially from ineffective DNA repair mechanisms [5] and deregulated stem cell differentiation signals [6]. The repertoire of these intrinsic changes in preneoplastic TICs confers neoplastic features of uncontrolled proliferation, unlimited self-renewal, sustained angiogenesis, abnormal differentiation, and tissue invasion and metastasis making hallmarks of cancer [3]. These mutations act as drivers for transformation of cells primed with genetic and epigenetic changes to form preleukemic clones. The preleukemic clones proceed in evolution with additional mutations and clonal selection causing occult leukemias and solid tumors [7–9]. Sequencing studies revealed about 140 genes that when altered by intragenic mutations can act as driver mutations during tumorigenesis [10]. A typical tumor contains two to eight driver mutations, while the remaining are passenger mutations that do not directly confer selective advantages [10], but might play critical roles in orchestrating the genetic interactions between driver mutations and the microenvironment towards tumor progression. The numbers of driver and passenger mutations that were revealed by sequencing vary among tumor types and even from patient to patient. Genome sequencing studies have also found that pediatric leukemias harbor on average 9.6 mutations per tumor, while melanomas and lung cancer might harbor more than 200 mutations per tumor [10]. The latter might reflect effects of environmental factors such as smoking and UV radiation that play significant roles in the etiology of lung cancer and melanomas, respectively.

It is believed that leukemia and neoplasms in general are abnormal outgrowth from a TIC(s) whose progeny sequentially accumulate nested genetic and epigenetic mutations in cancer genes, over an extended period of time during clonal evolution [11], that generate cellular diversity [12] and clonal expansion [13–15]. TICs are the cellular drivers of clonal expansion that can predict aggressive disease, and the TICs stem cell signature was shown to influence leukemia clinical outcome [16]. TICs (sometimes referred to as Cancer Stem Cells (CSCs), however, the CSCs term may only be used when self-renewal potential is a recognized feature of the described cell type, i.e. some TICs may not be self-renewing stem cells) were initially revealed through transplantation of subfractions of leukemic cells in mice [17]. While the existence of CSCs is human tumors is still contentious, the evidence for a central role of TICs in tumor development and resistance to therapy was recently solidified by lineage tracing studies in mice [18–20]. Longitudinal tracking of single cell-derived tumor cell clonal formation and monitoring of tumor development from TICs are unfeasible at the current level of knowledge. Therefore, there has been no consensus on the frequencies of TICs in the same tumor types and whether they are rare or frequent cells within each tumor. Understandably, the frequencies of TICs are most likely dynamic variables that reflect changes during the different phases of each tumor growth and also in response to changes in the tumor microenvironment [21,22].

In addition to genetic drivers, non-genetic drivers of clonal tumor cell selection during clonal evolution are also recognized, and among them are activation of alternative signaling, cell quiescence and epigenetic drivers. The first of these non-genetic drivers of formation of dominant clones during clonal evolution include the activation of alternative or downstream signaling pathway through signaling plasticity to achieve growth advantage or resistance to targeted therapy, a phenomenon termed oncogenic bypass [23]. Additional mechanisms of selection and drug resistance that are developed by TICs during clonal evolution of therapy resistance clones include cell quiescence of tumor stem cells [24], whereas these cells remain dormant awaiting further activating signals. Cell quiescence or cell dormancy is a mechanism also employed by disseminated tumor cells that shed from primary tumors and may lie dormant in distant tissues for long periods of time while retaining their potential for clonal activation resulting in metastatic growth [25]. Epigenetic changes affecting DNA methylation or chromatin proteins are likely to exert major influences on clonal evolution by mediating abnormal DNA methylation, histone modifications and/or nucleosome remodeling [26]. Cancer epigenome revealed simultaneous global losses and abnormal gains in DNA methylation. The rates of these epigenetic changes are estimated to be significantly higher than gene mutations [27]. However, unlike genetic changes in the DNA, DNA methylation varies between different cell types, developmental stages, and with aging [28]. Moreover, criteria for distinguishing epigenetic changes that would represent driver changes and provide selective advantages during clonal evolution are less established. Additionally, targeting epigenetic drivers with current and future epigenetic cancer therapy depends on adjusting treatment concepts towards dose optimization to avoid widespread toxicities [29]. The progression of clonal evolution of cancer, driven by genetic and non-genetic drivers from frequent preneoplastic lesions, is a highly inefficient process, and is frequently aborted before formation of occult malignancies [30]. When genetic sequences from multiple subclones were compared using single cell sequencing, the branched clonal structures of these cancers were revealed [31]. Furthermore, the evolutionary relationship and clonal architecture of Acute Lymphoblastic Leukemia (ALL) examined in stem cell populations demonstrated repeated independent copy number alteration within the same leukemic tumors [32]. Evidence for clonal evolution from ancestral clones was revealed earlier in non-Hodgkin’s lymphoma [33], and more recently in BCR-ABL1 ALL [34], ALL in twins [32], T-cell ALL [35], and during leukemic relapse [36]. Therefore, mapping and targeting tumor clones without driving additional clonal evolution and/or development of resistant clones is an optimum goal for cancer therapy.

## Tumor Heterogeneity

Tumor heterogeneity is recently recognized in leukemias and solid tumors [37], and is attributed to multiple levels of heterogeneity at the cellular, molecular, genetic, and therapeutic response levels. Variations between tumors arising at the same site accounts for intertumoral heterogeneity, while variations in clonal growth, functional properties or expression markers delineate intratumoral heterogeneity. Genetic heterogeneity between different tumor clones and even within the same clones is a common feature in many tumor types [38]. Moreover, cells within single genetic clones were deemed to display functional variability in tumor propagation potential when single lentivirus-marked lineages were examined for copy number alterations, sequencing, and lentiviral lineage tracking [39]. This study revealed another layer of functional complexity beyond the genetic heterogeneity that drives the intratumoral heterogeneity, and might define responses to therapy (Figure 1).

The recognition that driver mutations frequently encode protein kinases has led the recent use of EGFR inhibitors [40], ALK inhibitors [41], BRAF inhibitors [42], and PARP inhibitors [43] in cancers that are proven to harbor these mutations, or their signaling pathway such as BRCA gene mutations in the case of PARP inhibitors. Combination therapy utilizing these and other newer drugs that target multiple components of driver signaling pathways should be tailored to fit the genetic interactions [44] within individual genomic repertoire of each patient in personalized or precision medicine approach. Alternative approaches may target the TICs, the microenvironment, or to keep tumors clinically manageable without selection of resistance clones utilizing modified dosing regimen and intermittent therapy. Addressing the key challenges of defining the order of events during tumor progression, the roles of driver and/or passenger mutations in clonal selection and heterogeneity, and mechanisms of development of therapy resistant clones will lead to significant advances in cancer care.
